# An assessment of hypercapnia-induced elevations in regional cerebral perfusion during combined orthostatic and heat stresses

**DOI:** 10.1186/s12576-020-00751-4

**Published:** 2020-05-04

**Authors:** Manabu Shibasaki, Kohei Sato, Ai Hirasawa, Tomoko Sadamoto, Craig G. Crandall, Shigehiko Ogoh

**Affiliations:** 1 Department of Health Sciences, Faculty of Human Life and Environment, Nara Women’s University, Kitauoya-Nishi Machi, Nara, 630-8506 Japan; 2 Department of Health and Physical Education, Tokyo Gakugei University, Tokyo, Japan; 3 Department of Health and Welfare, Kyorin University, Tokyo, Japan; 4 Research Institute of Physical Fitness, Japan Women’s College of Physical Education, Tokyo, Japan; 5 Institute for Exercise and Environmental Medicine, Texas Health Presbyterian Hospital, Dallas, USA; 6 Department of Internal Medicine, University of Texas, Southwestern Medical Center, Dallas, USA; 7 Department of Biomedical Engineering, Toyo University, Saitama, Japan

**Keywords:** Orthostatic tolerance, Presyncope, Hyperthermia, Cerebral blood flow, CO_2_ reactivity

## Abstract

We investigated that the effects of hypercapnia-induced elevations in cerebral perfusion during a heat stress on global cerebrovascular responses to an orthostatic challenge. Seven volunteers completed a progressive lower-body negative pressure (LBNP) challenge to presyncope during heat stress, with or without breathing a hypercapnic gas mixture. Administration of the hypercapnic gas mixture increased the partial pressure of end-tidal CO_2_ greater than pre-heat stress alone, and increased both internal carotid artery (ICA) and vertebral artery (VA) blood flows (*P* < 0.05). During LBNP, both ICA and VA blood flows with the hypercapnic gas mixture remained elevated relative to the control trial (*P* < 0.05). However, at the end of LBNP due to pre-syncopal symptoms, both ICA and VA blood flows decreased to similar levels between trials. These findings suggest that hypercapnia-induced cerebral vasodilation is insufficient to maintain cerebral perfusion at the end of LBNP due to pre-syncope in either the anterior or posterior vascular beds.

## Introduction

Passive heat stress reduces orthostatic tolerance [1, 2], but the physiological mechanism(s) responsible for this occurrence remains debatable. A primary mechanism leading to pre-syncopal symptoms is thought to cerebral hypoperfusion [3]. Head-up tilt or lower body negative pressure (LBNP) are often used to investigate physiological responses to orthostatic-induced fluid shifts in humans [4]. Hyperthermia associated with passive heat stress induces hyperventilation and associated hypocapnia-induced reductions in cerebral perfusion [5, 6]. Such hyperthermia-induced cerebral hypoperfusion may contribute to heat-induced reductions in orthostatic intolerance [2, 5]. Since cerebral perfusion can be modulated through changes in arterial carbon dioxide tension (PaCO_2_) [7], it may be that heat-induced orthostatic intolerance can be prevented by inhaling a hypercapnic gas mixture prior to the orthostatic challenge.

Indeed, Howden et al. demonstrated that the inhalation of a hypercapnic gas mixture improved orthostatic tolerance, simulated by LBNP, in normothermic subjects [8]. Given these findings, Lucas et al. addressed the same question but in heat-stressed subjects. In that study, cerebral perfusion was estimated by transcranial Doppler-derived middle cerebral artery blood velocity (MCA_vel_) [9]. Despite substantially elevated MCA_vel_ secondary to inhalation of the hypercapnic gas mixture, LBNP tolerance was not improved in heat-stressed individuals relative to the trial without administering the hypercapnic gas mixture. However, a limitation of that protocol is the possibility that MCA_vel_ was not representative of changes in global cerebral perfusion (i.e., MCA_vel_ primary reflects blood flow to the anterior regions). Lewis et al. reported no difference between MCA and posterior cerebral artery (PCA) blood velocities during a normothermic orthostatic challenge [10]. In contrast, we observed different volumetric blood flow responses between the internal carotid artery (ICA) and the vertebral artery (VA) during progressive orthostatic stress, with VA blood flow being maintained while ICA blood flow decreased [11, 12]. Importantly, the regulation between ICA and VA blood flows during heat stress is different from that observed in normothermia [13].

Therefore, it is possible that cerebral perfusion in the posterior regions, supplied by the basilar and VA arteries, respond differently during combined heat and orthostatic stresses, relative to anterior regions (e.g., regions supplied by the MCA). Notably, such responses are speculative without volumetric assessments of regional blood flow during such stressors. We, therefore, hypothesized that the relative changes in volumetric cerebral blood flow between the anterior (ICA) and posterior cerebral arteries (VA) are different during combined heat and orthostatic stresses, and such differences are modified by a hypercapnic challenge (i.e., the inhalation of a hypercapnic gas mixture attenuates the decrease in cerebral blood flow in the anterior but not posterior regions).

## Methods

### Ethical approval

Experimental procedures and the protocol conformed to the Declaration of Helsinki and were approved by the Human Subjects Committee of Nara Women’s University (14-01). Each subject provided written, informed consent after all potential risks and procedures were explained.

### Subjects

Fourteen subjects enrolled in this study, but only ten completed both limbs of the protocol. Of those ten subjects, analyzable data were obtained from seven subjects due primarily to an inability to obtain images of sufficient quality at the termination of lower body negative pressure (LBNP) in three subjects. These seven subjects (four men and three women) had a mean ± SD age of 21 ± 1 years, height of 163 ± 9 cm and weight of 57 ± 14 kg. The subjects were free of any known cardiovascular or pulmonary disorders and were not using any prescribed or over-the-counter medications. In addition, subjects did not engaged in endurance training on a regular basis (< 5 h/week). For female subjects, repeated testing was conducted at the same phase of the menstrual cycle, although menstrual cycle phase was not controlled. Subjects abstained from caffeinated beverages for 12 h, and from strenuous physical activity and alcohol for at least 24 h, before each trial.

### Experimental measurements

At each experimental day, subjects emptied their bladder before being weighed to identify nude body mass. Subjects, wearing short pants and underwear, were instrumented with ECG and skin temperature probes, and then dressed in a long-sleeved and long-legged, two-pieced, tube-lined perfusion suit (Med-Eng, Ottawa, Canada) enabling the control of skin temperature and internal body temperature via changing the water temperature perfusing the suit. The suit covered the entire body, except for the head, face, hands, one arm, and feet. As index of internal body temperature, external canal temperature (*T*_ca_) was measured using an infrared temperature probe (CE Thermo, NIPRO Inc., Osaka, Japan). The validity of this measure was previously confirmed against esophageal temperatures [14, 15]. Whole-body mean skin temperature (*T*_sk_) was measured from the electrical average of six thermocouples [16] fixed to the skin with porous tape. Heart rate (HR) was obtained from an electrocardiogram (Biomulti 1000, NEC, Tokyo, Japan) and intermittent arterial blood pressure was obtained by auscultation of the brachial artery via electrosphygmomanometry (STBP-780, Colin, Tokyo, Japan). Beat-to-beat arterial blood pressure from a finger cuff was measured and reconstructed to give brachial artery pressures (Portapress, FMS, Amsterdam, The Netherlands). Finger arterial pressure was used solely to aid in the detection of presyncope during LBNP, while measures from the brachial artery were used for data analysis. Thermal and hemodynamic data were acquired continuously at 50 Hz throughout the experiment (MP150, Biopac, Santa Barbara, CA, USA). The subject breathed through a mouthpiece attached to a low-resistance two-way valve and pressure flowmeter. The valve mechanism allowed the subjects to inspire either room air or a hypercapnic gas mixture (5% CO_2_, 21% oxygen, balanced nitrogen) from a 200-L Douglas bag. Gas samples were obtained at the mouthpiece from which PetCO_2_ was obtained. Respiratory variables throughout the experiment were recorded by an automatic breath-by-breath respiratory gas analyzing system (ARCO2000 MET; Arcosystem, Chiba, Japan).

#### Doppler measurements

The right ICA and left VA diameters and blood velocities were measured with two ultrasound systems (Vivid i; GE Healthcare, Tokyo, Japan), each equipped with a 10-MHz linear transducer, from which volumetric blood flows were calculated. Right ICA measurements were performed ~ 1.0–1.5 cm distal to the carotid bifurcation while left VA measures was obtained between the transverse processes of C5 and the subclavian artery. The systolic and diastolic diameters were measured from three cardiac cycles in each stage, from which the mean diameter (in centimeters) was calculated as:

$${\text{Mean}}\;{\text{diameter}} = \left( {{\text{systolic}}\;{\text{diameter}} \times {1 \mathord{\left/ {\vphantom {1 3}} \right. \kern-0pt} 3}} \right) + \left( {{\text{diastolic}}\;{\text{diameter}} \times {2 \mathord{\left/ {\vphantom {2 3}} \right. \kern-0pt} 3}} \right).$$

The time-averaged mean blood flow velocities were recorded and analyzed (in centimeters per second). Blood flow velocity measures were obtained from the average of ~ 10–20 cardiac cycles, to account for any effects caused by respiration. Subjects were excluded if we were unable to obtain velocity recordings for a minimum of 4 continuous cardiac cycles of sufficient quality that were analyzable throughout each perturbation. Great care was taken to ensure that the probe position was stable, the insonation angle did not vary (60 deg in most cases), and the sample volume was positioned in the center of the vessel and adjusted to cover the width of the vessel diameter. Finally, volumetric blood flow was calculated by multiplying the cross-sectional area [*π* × (mean diameter/2)^2^] by mean blood flow velocity, as follows:

$${\text{Blood}}\;{\text{flow}} = {\text{mean}}\;{\text{blood}}\;{\text{flow}}\;{\text{velocity}} \times {\text{area}} \times 60\left( {{{\text{mL}} \mathord{\left/ {\vphantom {{\text{mL}} {\text{mintue}}}} \right. \kern-0pt} {\text{mintue}}}} \right).$$

### Experimental protocol

Subjects reported to the laboratory on two separate occasions. With each visit, subjects underwent a progressive LBNP trial while heat stressed either with or without inhalation of the hypercapnic gas mixture. The order of the experimental trials was randomized, and separated by a minimum of 3 days, with each trial being performed at the same time of day. After instrumentation, subjects were positioned in the LBNP box that was sealed at the level of the iliac crest. Subjects rested supine for at least 30 min prior to normothermic measurements (Norm baseline). Subjects were then passively heated by circulating warm water (~ 50 °C) through the suit until *T*_ca_ increased by ~ 1.2 °C. Then, water temperature was slightly decreased to temper the rate of rise in *T*_ca_. When *T*_ca_ was stable (~ 1.4 °C above Norm baseline), baseline heat-stressed measurements (Heat baseline) were obtained prior to the onset of LBNP. Subsequently, beginning at 20 mmHg, 3-min stages of LBNP were applied at 10 mmHg per stage until the occurrence of syncopal symptoms. In the CO_2_ trial, subjects inhaled the hypercapnic gas mixture 2 min before the onset of the LBNP challenge, with 1 min of hypercapnic data (Heat CO_2_) collected before the onset of LBNP. The LBNP challenge was then applied with the subject continuing to breathe the hypercapnic gas mixture or room air throughout. Test termination was based on the following criteria: continued self-reporting by the subject of feeling faint or feeling like he/she could no longer tolerate LBNP, pallor, diaphoresis, rapid and progressive decrease in blood pressure resulting in systolic blood pressure being less than 70 mmHg, and/or relative bradycardia accompanied with narrowing of pulse pressure. Typically, a combination of the aforementioned conditions was observed at the cessation of the tolerance test, with a reduction in pulse pressure along with relative bradycardia being the most common observations. The total time of each LBNP challenge was measured and used to determine a cumulative stress index (CSI) by summing the product of the negative pressure and duration at each stage of LBNP, in minutes and fraction of minutes (e.g., 20 mmHg × 3 min + 30 mmHg × 3 min + 40 mmHg × 3 min, etc.) until the test was terminated [17].

### Data analysis

In both trials, 60 s of data were averaged for Norm baseline before the start of whole-body heating. Heat baseline values were averaged from 60 s of data after *T*_ca_ had increased by ~ 1.4 °C. Thermal (i.e., *T*_sk_ and *T*_ca_), hemodynamic (heart rate; HR and mean arterial pressure; MAP), and respiratory (minute ventilation; VE, end-tidal carbon dioxide partial pressure P_ET_CO_2_, and respiratory rate; RR) variables before application of the hypercapnic gas mixture were evaluated by a two-way repeated measures analysis of variance (ANOVA) with main factors of time (Norm baseline vs. Heat baseline) and treatment days. In the CO_2_ trial, the hypercapnic data (Heat CO_2_) were evaluated by paired T test (Heat baseline vs. Heat CO_2_) to confirm the effect of the hypercapnic gas on the assessed variables. For the analyses of the responses to the LBNP challenge, Pre-LBNP data (i.e., prior to the LBNP challenge) were used from Heat baseline for the Air trial while Heat CO_2_ baseline data were used for the CO_2_ trial. The LBNP analysis included averaged responses during the last 12 s before the occurrence of any pre-syncopal symptoms (primarily the point just before a reduction in heart rate during the LBNP challenge; termed severe LBNP). Tolerance data were obtained by averaging the responses during the last 12 s before the end of the LBNP challenge due to syncopal symptoms. A three-way repeated measures ANOVA with main factors of region (ICA vs. VA), time (pre-LBNP, severe LBNP, end of LBNP) and experimental day (Air vs. CO_2_) was used only to compare relative changes in ICA and VA blood flows during the LBNP challenge. Two-way repeated measures ANOVA with main factors of time (pre-LBNP, severe LBNP, end of LBNP) and experimental day (Air vs. CO_2_) were used to identify differences in the assessed variables between the Air and CO_2_ trials. Any main effect differences were further explored via Student–Newman–Keuls post hoc tests. ICA and VA blood flow were normalized relative to the respective normothermic baselines, thereby enabling a comparison of regional differences. Paired *t* tests were used to identify differences in CSI between Air and the CO_2_ trials. The *α* level for all analyses was set at 0.05. Results are reported as mean ± SD.

## Results

### Physiological responses prior to the LBNP challenge

Pre-heat stress thermal, hemodynamic, and respiratory baseline measures were not difference between the trials (Table 1). Heat stress increased *T*_sk_ similarly between trials (Table 1) and increased *T*_ca_ by 1.40 ± 0.07 °C (Air trial) and 1.41 ± 0.14 °C (CO_2_ trial). Absolute and relative reductions in body mass as a result of the heat stress were similar between trials (Air trial: 56.7 ± 14.1 to 55.9 ± 13.9 kg; CO_2_ trial: 56.9 ± 14.3 to 56.0 ± 14.1 kg). Changes in hemodynamic and respiratory variables as a result of the heat stress were similar between these trials prior to inhaling the hypercapnic gas mixture (Table 1). During heating, absolute VA blood flow was slightly higher in the Air trial relative to the CO_2_ trial, but normalized responses (i.e., %baseline data) were similar between trials. Heat stress decreased ICA, but not VA, blood flows. Inhaling the hypercapnic gas mixture increased ICA and VA blood flows by ~ 1.3-fold relative to the respective normothermic baseline values, increased respiratory responses, while temperatures, HR and MAP were maintained (Table 1).

**Table 1:** Thermal, hemodynamic, respiratory variables, and blood flows

	Condition	Stage			ANOVA			*T* test
		Norm baseline	Heat baseline	Heat–CO_2_	Condition	Stage	Interaction	
*T*_sk_, °C	Air	33.8 ± 0.7	39.0 ± 0.7	38.9 ± 1.0	0.959	< 0.001	0.471	0.724
	CO_2_	33.9 ± 0.5	38.9 ± 1.0					
*T*_ca_, °C	Air	37.2 ± 0.3	38.6 ± 0.2	38.7 ± 0.1	0.775	< 0.001	0.365	0.060
	CO_2_	37.3 ± 0.1	38.6 ± 0.1					
HR, bpm	Air	61.7 ± 7.1	104.3 ± 11.9	102.6 ± 8.7	0.083	< 0.001	0.128	0.067
	CO_2_	60.4 ± 7.5	98.3 ± 7.4					
MAP, mmHg	Air	76.4 ± 8.5	77.9 ± 8.9	86.3 ± 8.7	0.207	0.717	0.171	0.045
	CO_2_	85.1 ± 14.4	82.0 ± 9.1					
VE, L/min	Air	7.1 ± 1.5	9.3 ± 2.8	17.0 ± 4.4	0.876	0.011	0.971	0.002
	CO_2_	7.3 ± 1.9	9.6 ± 2.0					
RR, *n*	Air	14.7 ± 2.4	15.9 ± 5.0	18.1 ± 5.9	0.227	0.309	0.894	0.414
	CO_2_	14.1 ± 2.6	16.4 ± 3.4					
P_ET_CO_2_, mmHg	Air	42.9 ± 2.6	40.0 ± 5.4	49.3 ± 3.6	0.876	0.054	0.902	0.002
	CO_2_	43.0 ± 2.4	40.3 ± 4.0					
VA, mL/min	Air	143.5 ± 43.8	141.8 ± 51.5	171.1 ± 51.7	0.045	0.439	0.619	0.010
	CO_2_	132.0 ± 41.2	123.0 ± 34.4					
ICA, mL/min	Air	398.2 ± 72.9	352.2 ± 87.8	459.8 ± 100.5	0.586	0.039	0.645	0.019
	CO_2_	369.8 ± 70.8	337.9 ± 73.0					
%VA, %baseline	Air	100	99.2 ± 18.7	133.0 ± 33.1	0.632	0.582	0.632	0.010
	CO_2_	100	94.7 ± 17.7					
%ICA, %baseline	Air	100	88.7 ± 16.2	127.0 ± 35.7	0.685	0.040	0.685	0.040
	CO_2_	100	91.5 ± 9.5					

Blood flow values are depicted in absolute units and relative changes from the normothermic baseline prior to LBNP challenge. Values are mean ± SD were averaged from steady-state supine data at normothermic (Norm baseline) and during heat stress (Heat baseline). Prior to the LBNP challenge, the hypercapnic gas was applied in the CO_2_ trial (Heat CO_2_) only. *T*_sk_, mean skin temperature; *T*_ca_, external canal temperature; VE, minute ventilation; RR, respiratory rate; P_ET_CO_2_, partial pressure of end-tidal carbon dioxide; MAP, mean arterial blood pressure; HR, heart rate; ICA, internal carotid artery; VA, vertebral artery blood flow. ICA and VA blood flows were depicted in absolute units and relative changes (i.e., normalized relative to the respective normothermic baselines). Subjects performed two progressive lower body pressure during heat stress with (CO_2_ trial) or without (Air trial) an application of hypercapnic gas mixture on different day. Thermal, hemodynamic, respiratory, and cerebrovascular variables were evaluated by a two-way repeated measures analysis of variance (ANOVA). The paired *T* test (Heat baseline vs. Heat CO_2_) performed to evaluate the effect of inhaling hypercapnic gas on these variables

### Responses to the LBNP challenge

**Fig. 1:**
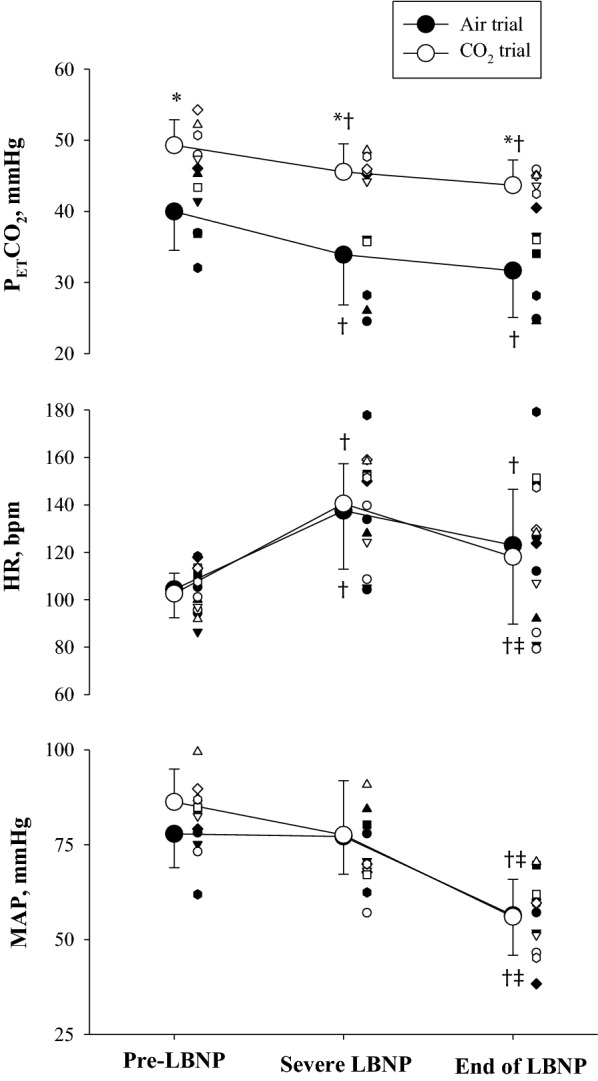
End-tidal carbon dioxide partial pressures (P_ET_CO_2_, top), heart rate (HR, middle), and mean arterial pressure (MAP, bottom) during both heat stress and LBNP where subjects inhaled either a hypercapnic gas mixture (CO_2_ trial) or room air (Air trial). In the CO_2_ trial subjects inhaled a 5% CO_2_ gas mixture prior to the onset of LBNP. Pre-LBNP indicates the responses prior to the LBNP challenge while heat stressed. Severe LBNP indicates the responses at the highest heart rate achieved during LBNP (i.e., prior to any bradycardia), End of LBNP indicates the responses at the end of LBNP. *, indicates difference from the Air trial (*P* < 0.05); † and ‡, indicate difference from Pre-LBNP, and Severe LBNP, respectively (*P* < 0.05). Individual data were plotted with different symbols

LBNP tolerance was similar between the trials (CSI for Air: 178.5 ± 114.5 mmHg × min vs. CSI for CO_2_: 269.5 ± 171.6 mmHg × min, *P* = 0.272). Consistent with that observation, the final LBNP stage achieved was also not different between trials (Air: 34 ± 10 vs. CO_2_: 44 ± 11 mmHg, *P* = 0.11). *T*_ca_ was maintained throughout LBNP in both the Air and CO_2_ trials (end of LBNP: 38.6 ± 0.3 °C vs. 38.6 ± 0.2 °C, respectively), while *T*_sk_ was slightly decreased for the CO_2_ trials (end of LBNP: 38.8 ± 0.8 °C vs. 38.6 ± 1.5 °C, respectively). The LBNP challenge increased HR, but MAP was maintained in both trials prior to the onset of pre-syncope symptoms (Fig. 1). At the end of LBNP, MAP was significantly decreased in both trials, with the magnitude of this reduction being similar between trials. P_ET_CO_2_ in the Air trial decreased to 31.7 ± 6.6 mmHg at the end of LBNP. For the CO_2_ trial, administration of the hypercapnic gas mixture increase P_ET_CO_2_ to above normothermic baseline, with P_ET_CO_2_ at the end of LBNP (43.7 ± 3.6 mmHg) being similar to P_ET_CO_2_ at normothermic baseline (43.0 ± 2.4 mmHg).

**Fig. 2:**
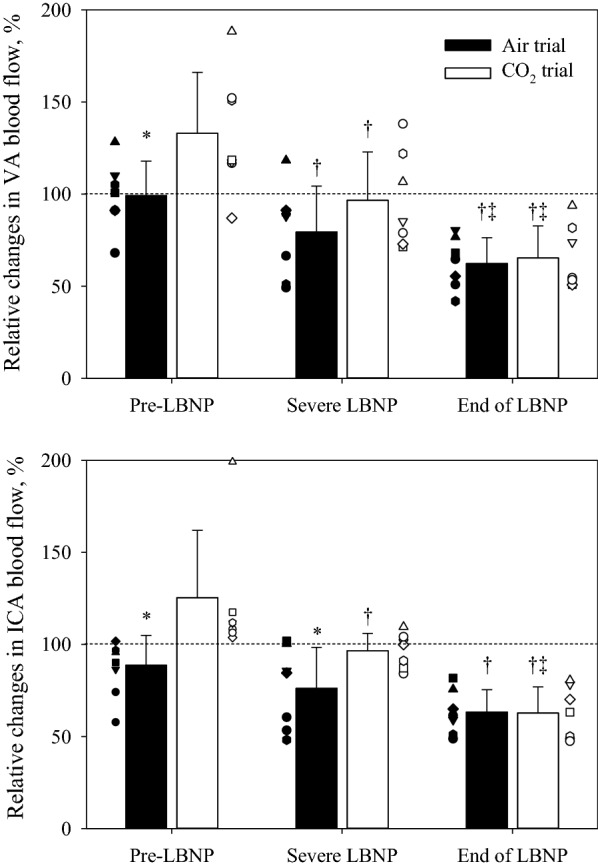
Relative changes in vertebral artery (VA, top) and internal carotid artery (ICA, bottom) blood flow at each LBNP stage (Pre-LBNP, Severe LBNP and End of LBNP), with (open) or without (solid) the hypercapnic gas. Application of the hypercapnic gas mixture increased both ICA and VA blood flows. LBNP decreased blood flow to both regions (i.e., Severe LBNP). However, at Severe LBNP in the hypercapnic trial, both ICA and VA blood flows were at a similar level relative to normothermic baseline (indicated by the dashed horizontal line), whereas in the Air trial those were values were below normothermic baseline. At the end of LBNP, due to presyncope, both ICA and VA blood flows were equivalent and both were lower than normothermic baseline. *P < 0.05 different from the Air trial; ^†^*P* < 0.05 different from Pre-LBNP; ^‡^*P* < 0.05 different from Severe LBNP

**Fig. 3:**
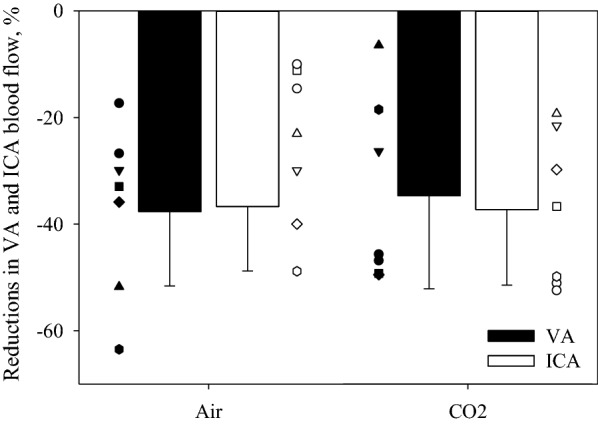
Reductions in relative changes of vertebral artery (VA, top) and internal carotid artery (ICA, bottom) blood flow to LBNP stress with (open) or without (solid) the hypercapnic gas. Both ICA and VA blood flows were decreased below normothermic baseline at the end of LBNP

ANOVAs for relative changes in blood flows during LBNP, with or without inhaling the hypercapnic gas mixture, showed a significant main effect of time and experimental day, but not for regional cerebral perfusion (see Figs. 2, 3 and Table 2). Figure 2 shows the relative changes in ICA and VA blood flows to the LBNP challenges. Before LBNP, both ICA and VA blood flows in the CO_2_ trial were elevated above the normothermic baseline and were both greater relative to the same time point in the Air trial. LBNP decreased both blood flows in each trial, and at the end of LBNP both ICA and VA blood flows had decreased below the respective normothermic baselines (dashed line in Fig. 2); this was accomplished by both diameters and velocities of ICA and VA decreasing at the end of LBNP relative to pre-LBNP during both Air and CO_2_ trials (Table 2). The relative reduction in ICA and VA blood flows to LBNP from the normothermic baseline were not different between trials, with ICA and VA blood flows decreasing to 63.3 ± 12.1% and 62.4 ± 14.0% in the Air trial and to 62.7 ± 14.2% and 65.3 ± 17.5% in the CO_2_ trial, respectively. ANOVAs for the reductions in relative blood flows due to LBNP, with or without inhaling the hypercapnic gas mixture, showed no significant main effect of regions (*P* = 0.843), experimental day (*P* = 0.811), or interaction (*P* = 0.664). Thus, regardless of the inhaled gas, no regional differences between ICA and VA blood flows, normalized by normothermic baseline values, were observed (Fig. 3).

**Table 2:** Internal carotid artery (ICA) and vertebral artery (VA) responses during lower body negative pressure (LBNP)

	Condition	Stage			ANOVA		
		Pre-LBNP	Severe LBNP	End of LBNP	Condition	Stage	Interaction
VA blood flow, mL/min	Air	141.8 ± 51.5	116.8 ± 53.5^†^	92.2 ± 42.0^†‡^	0.465	< 0.001	0.102
	CO_2_	171.1 ± 51.7	124.8 ± 42.3^†^	85.5 ± 34.8^†‡^			
ICA blood flow, mL/min	Air	352.2 ± 87.8	302.5 ± 104.5	251.2 ± 66.2^†^	0.153	< 0.001	0.061
	CO_2_	459.8 ± 100.5*	356.9 ± 76.7^†^	229.6 ± 54.1^†‡^			
VA diameter, cm	Air	0.37 ± 0.05	0.36 ± 0.04	0.35 ± 0.05^†^	0.519	< 0.001	0.861
	CO_2_	0.36 ± 0.05	0.35 ± 0.04^†^	0.34 ± 0.05^†^			
ICA diameter, cm	Air	0.49 ± 0.05	0.47 ± 0.05	0.46 ± 0.04^†^	0.901	0.001	0.762
	CO_2_	0.49 ± 0.04	0.48 ± 0.05	0.46 ± 0.06^†^			
VA velocity, cm/s	Air	21.86 ± 3.06	18.45 ± 5.58^†^	15.50 ± 3.95^†^	0.297	< 0.001	0.063
	CO_2_	27.43 ± 6.24	21.75 ± 6.67^†^	15.39 ± 2.81^†‡^			
ICA velocity, cm/s	Air	31.66 ± 6.13	28.98 ± 8.11	24.61 ± 5.06^†^	0.206	< 0.001	0.043
	CO_2_	40.75 ± 6.91*	34.02 ± 10.6^†^	22.77 ± 4.52^†‡^			

Values are mean ± SD that were averaged from steady-state supine data pre-lower body negative pressure application (Pre-LBNP), just before the occurrence of any pre-syncopal symptoms (Severe LBNP), and at the end of the LBNP challenge due to syncopal symptoms (End of LBNP). ICA: internal carotid artery, VA: vertebral artery (VA). * *P* < 0.05, difference between Air and CO_2_; ^†^*P* < 0.05, difference from Pre-LBNP; ^‡^*P* < 0.05, difference from Severe LBNP

## Discussion

Global cerebral blood flow responses during an LBNP tolerance test in heat-stressed subjects were evaluated with and without the administration of a hypercapnic gas mixture. The main observations of this study are: (1) despite an increase in both VA and ICA blood flows via an inhaling a hypercapnic gas mixture while heat stressed, LBNP tolerance was not improved; (2) there was no regional difference in the magnitude of the relative reduction in cerebral perfusion between ICA and VA vessels during LBNP for either the Air and CO_2_ trials; (3) at the end of LBNP, both ICA and VA blood flows decreased below the normothermic baseline values regardless of the inhaled gas. These findings show that the application of a hypercapnic gas mixture increased both anterior and posterior cerebral blood flow during heat stress, but this stimulus was insufficient to maintain cerebral perfusion at presyncope.

Excessive increases of core/internal temperature due to passive heat stress greatly reduce orthostatic tolerance [1, 2]. Passive heating decreases total peripheral vascular resistance, and also promotes hyperventilation-induced hypocapnia, particularly when core/internal temperature increase over ~ 1.0 °C, resulting in cerebral hypoperfusion [18–20]. Since cerebral hypoperfusion leads to pre-syncope symptoms [3], it was previously hypothesized that maintaining cerebral perfusion via inhaling hypercapnic gas in heat-stressed individuals would improve orthostatic intolerance [9]. However, Lucas et al. did not observe such a response, as elevating MCA_vel_ via inhaling hypercapnic gas did not alter LBNP tolerance in heat-stressed individuals [9]. They concluded that preserving cerebral perfusion does not influence orthostatic tolerance during hyperthermic condition. Consistent with the observations of Lucas et al., we also did not see differences in LBNP tolerance between trials (i.e., with or without the inhaling hypercapnic gas), despite P_ET_CO_2_ being maintained at or above pre-heat level throughout LBNP in the CO_2_ trial [9]. However, the unique aspect of the present study is the assessment of regional cerebrovascular beds both to the hypercapnic challenge while heat stressed, and throughout subsequent LBNP to tolerance.

Blood supply to the brain originates not only from the ICA, but also the VA, with ~ 65–70% of global cerebral blood flow being supplied by the ICA and the residual ~ 25–30% being supplied by the VA [21]. The ICA provides blood to the anterior cerebral circulation through the MCA and anterior cerebral artery, while the VA merges with the basilar artery to provide blood to the posterior cerebral circulation, including the medulla oblongata, hypothalamus, thalamus, brainstem and cerebellum [22]; regions that are recognized to control autonomic function. In normothermia, we previously observed that orthostatic stress decreases ICA blood flow, while VA blood flow is generally maintained in tolerant subjects (i.e., no observation of pre-syncope symptoms) [11, 23]. Although speculative, given these previous observations, coupled with no regional difference in cerebral perfusion in pre-syncope subjects during heat stress, further investigation is needed to clarify the rationale of regional cerebral perfusion in orthostatic tolerance.

Previously, we also observed that cerebrovascular CO_2_ reactivity in the VA was smaller than that in the ICA, regardless of thermal status [13]. Given those observations, we expected differential changes in VA and ICA blood flows to the imposed challenges in the present study, culminating in VA blood flow in the CO_2_ trial not being maintained (or maintained less) relative to ICA blood flow during combined heat and LNBP stress. Contrary to that hypothesis, both VA and ICA blood flows decreased during the LBNP challenge, and culminated in a similar relative reduction in ICA and VA blood flows at the end of LBNP, regardless of the administration of the hypercapnic gas mixture. Nevertheless, these results suggest that volumetric changes in global cerebral perfusion are important when assessing orthostatic tolerance in the heat-stressed individuals.

A previous study reported that MCA_vel_ remained greater than normothermic baseline at the end of LBNP during a hypercapnic challenge of heat-stressed individuals [9], which is inconsistent with our results. Recent findings raise a question about the reliability of transcranial Doppler-derived indices of CBF because the diameter of MCA can change with substantial changes in PaCO_2_ [24, 25]. Also, van Helmond indicated that MCA_vel_ during combined stresses, such as LBNP under hypoxia, might underestimate CBF because these stresses might change MCA diameter [26]. Therefore, the present protocol evaluated cerebral perfusion via volumetric changes in flow from regions perfused by both the ICA and VA, culminating in the observation that blood flows to both regions decreased below the respective normothermic baselines at the end of LBNP regardless of inhaled gas. Of note, we observed that the diameter of both the ICA and VA decreased during LBNP in both trials, while the velocities also decreased. Thus, under the imposed combined stresses, hypercapnia-induced increases in cerebral blood flow, due to cerebral vasodilation, is apparently overridden by reductions in perfusion pressure and/or accompanying cerebral vasoconstriction resulting in global decreases in cerebral blood flow. These findings suggest that regional cerebral perfusion cannot be maintained during combined heat and orthostatic stresses despite cerebral blood flow being elevated via the inhalation of a hypercapnic gas mixture.

There are two possible mechanisms for the observed heat stress-induced cerebral hypoperfusion, that is hypocapnia and hypotension [6, 10]. Either breathing a hypercapnic gas mixture or expansion of blood volume can attenuate cerebral hypoperfusion during a hypotensive episode. Based upon the present results and Lucas et al.’s prior observation [9], an elevated cerebral perfusion due to breathing a hypercapnic gas mixture is unlikely to improve orthostatic tolerance in hyperthermic individuals. In contrast, acute volume expansion improves orthostatic tolerance in hyperthermic subjects [27], while a follow-up study demonstrated that such acute volume expansion attenuates the reduction in cerebral perfusion during combined heat and orthostatic stresses [28]. These observations suggest that in a case of acute dehydration, acute volume expansion is capable of improving orthostatic tolerance perhaps by preserving cerebral perfusion. However, a recent study investigated the interactive effects of incremental heat stress, orthostatic stress, and hypohydration, and suggested that hypohydration before heat stress does not influence the cerebrovascular or cardiovascular responses to LBNP [29]. Therefore, the mechanism(s) of orthostatic intolerance in the heat-stressed human remains unknown.

### Limitation of this study

A previous study reported that MCA_vel_ remained greater at the end of LBNP due to syncopal symptoms [9], whereas ICA blood flow in this study was decreased below the normothermic baseline. Despite administration of the hypercapnic gas, P_ET_CO_2_ decreased slightly during LBNP, but this value did not decrease below P_ET_CO_2_ at normothermic baseline. Though such reductions in P_ET_CO_2_ might decrease ICA blood flow, P_ET_CO_2_ at the end of LBNP was significantly greater in the CO_2_ trial relative to the Air trial. Nevertheless, absolute ICA blood flow in the CO_2_ trial was similar with that in the Air trial at the end of LBNP, indicating that regardless of inhaled hypercapnic gas mixture ICA blood flow is not preserved during a profound orthostatic stress. Blood flow evaluation by duplex ultrasonography has the advantage of allowing simultaneous quantification of the vessel diameter and the blood velocity (i.e., volumetric assessment), which cannot be obtained by using the transcranial Doppler (TCD) measurement technique. However, given that continuous volumetric flow measures is challenging, particularly at low perfusion states such as LBNP, we could not ascertain the dynamics of ICA and VA blood flow responses precisely at the onset of presyncope. Also, VA and ICA ultrasound measures were performed on the left and right side, respectively, recognizing that the left VA blood flow and diameter are slightly greater relative to the right VA blood flow and diameter [12]. This approach was selected to prioritize obtaining clear blood flow waveforms over assessing vessels ipsilaterally. Another approach to evaluate the control of the cerebral vasculature is to perform transfer function analyses (TFA) between changes in cerebral perfusion and arterial blood pressure. However, TFA was not possible in the present trial due to the short time period of each LBNP state [30]. Finally, 14 volunteers participated in this study, with 10 subjects completing both LBNP trials, and just 7 providing analyzable data. Such a small sample size might result in beta errors in some results that were found to not be statistically significant.

## Conclusion

Global cerebral perfusion during LBNP in heat-stressed subjects was evaluated by volumetric changes of ICA and VA blood flows using duplex ultrasonography. An administration of hypercapnic gas increased blood flows through both vessels. Regardless of the inhaled hypercapnic gas mixture, ICA and VA blood flows decreased below their respective normothermic baselines at the end of LBNP, when pre-syncopal symptoms were evident. Thus the present results confirm that breathing hypercapnic gas mixture will not prevent orthostatic intolerance in the hyperthermic individuals. Moreover, regional difference in cerebral perfusion was not observed at the end of LBNP, regardless of whether PaCO_2_ is elevated, and global indices of cerebral perfusion were not maintained during severe orthostatic stress when combined with a heat stress.

## Data Availability

All relevant data are within the paper.

## Abbreviations

LBNP: Lower-body negative pressure
ICA: Internal carotid artery
VA: Vertebral artery
MCA: Middle cerebral artery
PCA: Posterior cerebral artery
MCA_vel_: Middle cerebral artery blood velocity
*T*_ca_: External canal temperature
*T*_sk_: Mean skin temperature
HR: Heart rate
MAP: Mean arterial blood pressure
PaCO_2_: Arterial carbon dioxide tension
CSI: Cumulative stress index
